# Azacytidine mitigates experimental sclerodermic chronic graft-versus-host disease

**DOI:** 10.1186/s13045-016-0281-2

**Published:** 2016-07-04

**Authors:** Gilles Fransolet, Grégory Ehx, Joan Somja, Loïc Delens, Muriel Hannon, Joséphine Muller, Sophie Dubois, Pierre Drion, Jo Caers, Stéphanie Humblet-Baron, Philippe Delvenne, Yves Beguin, Giuseppina Conteduca, Frédéric Baron

**Affiliations:** Groupe Interdisciplinaire de Génoprotéomique Appliquée (GIGA)-I3, Laboratory of Hematology, University of Liège, Liège, Belgium; GIGA-R, Department of Pathology, University of Liège, Liège, Belgium; GIGA-R, Animal care unit, University of Liège, Liège, Belgium; Department of Medicine, Division of Hematology, CHU of Liège, Liège, Belgium; Translational Immunology Laboratory, VIB, Leuven, Belgium; Department of Microbiology and Immunology, KU Leuven-University of Leuven, Leuven, Belgium; Department of Hematology, University of Liège, CHU Sart-Tilman, 4000 Liège, Belgium

**Keywords:** AZA, Azacytidine, Decitabine, DAC, Treg, Regulatory T cells, GVHD, Graft-versus-host disease, Chronic graft-versus-host disease, Sclerodermic graft-versus-host disease

## Abstract

**Background:**

Previous studies have demonstrated that regulatory T cells (Tregs) play a protective role in the pathogenesis of chronic graft-versus-host disease (cGVHD). Tregs constitutively express the gene of the transcription factor *Foxp3* whose CNS2 region is heavily methylated in conventional CD4^+^ T cells (CD4^+^Tconvs) but demethylated in Tregs.

**Methods:**

Here, we assessed the impact of azacytidine (AZA) on cGVHD in a well-established murine model of sclerodermic cGVHD (B10.D2 (H-2d) → BALB/cJ (H-2d)).

**Results:**

The administration of AZA every 48 h from day +10 to day +30 at the dose of 0.5 mg/kg or 2 mg/kg mitigated chronic GVHD. Further, AZA-treated mice exhibited higher blood and thymic Treg frequencies on day +35, as well as higher demethylation levels of the *Foxp3* enhancer and the *IL-2* promoter in splenocytes at day +52. Interestingly, Tregs from AZA-treated mice expressed more frequently the activation marker CD103 on day +52. AZA-treated mice had also lower counts of CD4^+^Tconvs and CD8^+^ T cells from day +21 to day +35 after transplantation, as well as a lower proportion of CD4^+^Tconvs expressing the Ki67 antigen on day +21 demonstrating an anti-proliferating effect of the drug on T cells.

**Conclusions:**

Our results indicate that AZA prevented sclerodermic cGVHD in a well-established murine model of cGVHD. These data might serve as the basis for a pilot study of AZA administration for cGVHD prevention in patients at high risk for cGVHD.

**Electronic supplementary material:**

The online version of this article (doi:10.1186/s13045-016-0281-2) contains supplementary material, which is available to authorized users.

## Background

Despite its association with graft-versus-tumor effects [1–6], chronic graft-versus-host disease (cGVHD) remains an important limitation of allogeneic hematopoietic cell transplantation (allo-HCT) [7–9]. CGVHD manifestations can be remarkably polymorphic, ranging from isolated lichenoid cunatenous and/or oral mucosa lesions to dramatic multi-systemic disease mimicking severe forms of systemic lupus erythematosus or systemic sclerosis [8, 10].

Recent progresses have been made in our understanding of cGVHD pathogenesis [10, 11]. It is now well-demonstrated that clinical manifestations of cGVHD in humans are mediated by a complex immune reaction involving donor conventional CD4^+^ and CD8^+^ T cells, regulatory T cells (Tregs), B cells, and regulatory B cells [10, 12, 13]. However, the beneficial effect of in vitro [14, 15] or in vivo [6, 16–18] T-cell depletion of the graft at preventing cGVHD demonstrates a primary role for donor T cells in cGVHD pathogenesis.

Regulatory T cells (Tregs) represent a fraction of CD4^+^ T cells that is indispensable for maintaining immunological self-tolerance [19, 20]. Tregs constitutively express the forkhead box protein 3 factor (Foxp3) in their nuclei, and CD25 (the high-affinity component of the trimeric form of the interleukin 2 (IL-2) receptor) on their cell surface [19, 20]. Consequently, IL-2 is the key cytokine for Treg homeostasis [20, 21]. Interestingly, an altered Treg homeostasis has been observed in patients with cGVHD [22–25], while transfer of activated (CD103^+^) Tregs ameliorated cGVHD in a mouse-to-mouse model of cGVHD [26]. Further, remarkably, correction of Treg homeostasis by administration of low-doses of IL-2 induced clinical responses in approximately 50 % of patients suffering from steroid-refractory cGVHD [23].

Although forced expression of Foxp3 in conventional CD4^+^ T cells (CD4^+^Tconvs) confers them a suppressive activity [27], Foxp3 expression *per se* is not sufficient for stably maintaining Treg phenotype and suppressive functions [28]. Indeed, Treg-specific epigenetic changes (such as DNA hypomethylation) are also involved in controlling the expression of some Tregs function-associated molecules such as IL-2ra, CTLA4, or Foxp3 itself [28]. Among these epigenetic changes, *Foxp3* conserved noncoding sequence 2 (CNS2) is unmethylated in Tregs while heavily methylated in CD4^+^Tconvs, and greatly contributes to the stability of Foxp3 expression as well as Treg phenotype and functions [28]. Further, the *Foxp3* enhancer is also demethylated in Tregs while heavily methylated in CD4^+^Tconvs [29]. Previous studies have demonstrated that hypomethylating agents (HMA) such as azacytidine (AZA) or decitabine (DAC) induce Foxp3 expression both in vitro and in vivo [30, 31] through the demethylation of *Foxp3* CNS2 region [30] and *Foxp3* enhancer [29].

Based on these findings, we herein investigated the impact of AZA on sclerodermic cGVHD (scl-cGVHD) in a classical murine model of scl-cGVHD (B10.D2 (H-2d) → BALB/cJ (H-2d)) [32–35].

## Methods

### Mice and drugs

Twelve- to 14-week-old B10.D2 (H-2d, Jackson Laboratories, Bar Harbor, USA) and BALB/cJ (H-2d, Jackson Laboratories) mice were used as donors and recipients, respectively. All mice were maintained in top-filtered cages in a standard animal facility and provided with sterilized food. Sterilized water, supplemented with 1 % Baytril (Bayer HealthCare, Diegem, Belgium), was given from day 0 to the end of the experiment. Water was changed every 2–3 days. All experimental procedures and protocols used in this investigation were reviewed and approved by the Institutional Animal Care and Use Ethics Committee of the University of Liège (Belgium), reference 1438. The “Guide for the Care and Use of Laboratory Animals”, prepared by the Institute of Laboratory Animal Resources, National Research Council, and published by the National Academy Press, was followed carefully as well as European and local legislation. 5-Azacytidine (AZA, Sigma-Aldrich, St. Louis, MO) or Decitabine (DAC, Dacogen, Janssen-Cilag, Zug, Switzerland), solubilized in PBS (Lonza, Verviers, Belgium), were injected s.c. every 48 h from D + 10 to D + 30 at the dose of 0.5 mg/kg or 2 mg/kg for AZA and 0.75 mg/kg for DAC. Control mice did not receive any injection.

### Cell preparation for bone marrow transplantation

Spleen, femurs and tibias from B10.D2 (or from BALB/cJ in case of syngeneic transplantation) mice were collected in sterile RPMI + 10 % FBS + 1 % penicillin/streptomycin. After red blood cell lysis (RBC lysis buffer; eBioscience, SD, USA), suspensions were passed through a 70 μm nylon filter mesh and washed with PBS + 3 % FBS + 1 % P/S. Cells were then resuspended and mixed in PBS at a concentration of 70 × 10^6^ spleen cells and 10 × 10^6^ bone marrow cells/200 μL and injected intravenously in recipient mice.

### Bone marrow transplantation and scl-cGVHD

BALB/cJ recipient mice were transplanted as previously described by Jaffee and Claman [32]. Briefly, recipients BALB/cJ mice (H-2d) first received a total body irradiation of 7 Gy (^137^Cs source, GammaCell 40, Nordion, Ontario, Canada). Five to six hours later, recipients were injected with 200 μL of the above described cell suspension. The severity of sclerodermatous cGVHD (scl-cGVHD) was assessed with a clinical scoring system as described earlier [35]. Briefly, ear-tagged animals were individually scored three times/week for five parameters: weight loss (grade 1, 10–20 %; grade 2, >20 %), posture (1, kyphosis only at rest; 2, severe kyphosis when the animal moves), activity (1, moderate activity impairment; 2, no move unless stimulated), skin (1, erythema or scaling tail; 2, open lesion on the body surface), and hair loss (1, > 1 cm^2^; 2, > 2 cm^2^). Clinical score was generated by summation of the five criteria scores (0–10/10). Animals reaching a score of 8/10 or a weight loss of >20 % of initial weight were sacrificed to avoid unnecessary pain according to our local ethic committee recommendations. Mice were observed every day for general health during the whole course of the experiments.

After 52 days, mice were sacrificed by anesthesia with 2 % Isoflurane (Forene®, Abbott Laboratories, Chicago, Illinois, USA) followed by cervical dislocation. Confirmation of death was done by monitoring absence of heart contraction or respiratory movements for 15 min. Splenocytes and bone marrow cells were collected in sterile RPMI + 10 % FBS + 1 % P/S. Lungs and skin were harvested in 10 % formaldehyde while blood and serum were collected in K2EDTA tubes and SST tubes, respectively (Becton Dickinson, Franklin Lakes, New Jersey, USA).

### Skin, lungs and thymus biopsies and immunohistochemistry

On day +29 after transplantation (two first cohorts of mice) or at sacrifice (day +52, third cohort of mice) skin samples of 0.5 cm^2^ from the upper back were obtained and directly fixed in 10 % formaldehyde for 48 h, embedded in paraffin and sectioned at 4–5 μm. Similarly, lungs (third cohort at day +52) and thymus (fifth cohort on day +35) were collected at sacrifice and directly fixed in 10 % formaldehyde for 48 h, embedded in paraffin and sectioned at 4–5 μm. Skin and lung sections were then stained with Goldner’s trichrome using Masson-Goldner staining kit following the manufacturer’s instructions (Merck Millipore, Darmstadt, Germany). Pulmonary fibrosis was assessed using the Ashcroft’s scale [36]. Thymic sections were stained with hematoxylin-eosin. For CD11b staining, paraffin-embedded sections were deparaffinized in xylene and rehydrated through a graded ethanol series. Antigen was retrieved with EDTA buffer combined with heating in a pressure cooker. Slides were peroxidase blocked with a commercial protein block serum-free solution (Dako, Glostrup, Danemark) for 10 min and were then incubated with the CD11b antibody (Abcam PLC, Cambridge, UK) during 1 h at room temperature and detected with the Envision system (Dako) for 30 min. Colorimetric detection was completed with diaminobenzidine (Dako) and slides were then counterstained with hematoxylin. Slides were then examined by a blinded examinator (JS) using a scoring system consisting in an absolute count of CD11b^+^ cells in three different hotspots, defined as areas in which CD11b staining is particularly high, for each slide. The total number of positive cells was then normalized to the number of CD11b^+^ cells per mm^2^. Histological slides were then scanned with a Hamamatsu Nanozoomer 2.0 HT (Hamamatsu Photonics, Geldern, Germany).

### Blood counts and flow cytometry

Blood was collected immediately after tail vein bleeding in BD Microtainer K2EDTA Tube (BD Biosciences, SD, USA). Splenocytes were obtained by crushing the spleen in RPMI 1640 + 10 % FBS. Blood cell populations were counted with a cell-dyn 3700 analyzer (Abbott Laboratories, Chicago, Illinois, USA). Erythrocytes were depleted using RBC lysis buffer (eBioscience) according to manufacturer’s instructions. Cells were washed in PBS + 3 % FBS (Lonza) before processing for flow cytometry. The following antibodies were used: anti-mouse CD3-V500 (500A2, Becton-Dickinson, Bedford, MA), anti-mouse CD3-PE (500A2), anti-mouse CD4-eFluor450 (RM4-5), anti-mouse CD4-APC-Cy7 (RM4-5, SONY Biotechnology, Weybridge, UK), anti-mouse CD8-PE-Cy7 (53–6.7), anti-mouse CD8-APC-eFluor780 (53–6.7), anti-mouse CD229.1-FITC (30C7, BD), anti-mouse CD25-APC (PC61.5), anti-mouse CD25-PE-Cy7 (PC61, BD), anti-mouse CD25-PercP-Cy5.5 (PC61.5), anti-mouse CD103-BV510 (M290, BD), anti-mouse CD90.2 APC (53–2.1) (all eBioscience unless indicated otherwise). Cells (1.5–2 × 10^6^ cells/sample) were incubated with surface antibodies for 20 min at 4 °C in the dark and washed twice with PBS + 3 % FBS. Intracellular staining for Foxp3 and Ki67 was performed by using the FoxP3 staining buffer set (eBioscience). For pSTAT5 staining, the PerFix EXPOSE reagent kit (Beckman Coulter, Fullerton, CA) was used following the manufacturer’s instruction as previously reported [37]. The following antibodies were used: anti-mouse Foxp3-APC (FJK-16 s, eBioscience), anti-mouse Foxp3-PE (FJK-16 s, eBioscience), anti-human phophoSTAT5-APC (pY694, BD) and anti-human Ki67-FITC (35/Ki-67, BD). Data were acquired on a FACS Canto II flow cytometer (Becton Dickinson) and analyzed with the FlowJo software 10.0.7 (Tree Star Inc., Ashland, OR). Cell phenotypes were defined as CD3^+^CD8^+^ for cytotoxic CD8^+^ T cells, CD3^+^CD4^+^Foxp3^-^ for CD4^+^Tconvs and CD3^+^CD4^+^Foxp3^+^ for Tregs. In the thymus, populations were described as follow: CD90.2^+^CD4^-^CD8^-^ for double negative (DN), CD90.2^+^CD4^+^CD8^+^ for double positive (DP), CD90.2^+^CD4^+^CD8^-^ for single CD4 positive, CD90.2^+^CD4^-^CD8^+^ for single CD8 positive, and CD90.2^+^CD4^+^CD8^-^Foxp3^+^ for Tregs. Absolute counts were calculated by multiplying the percentage of positive cells in the lymphoid gate by the absolute lymphocyte count measured by the cell-dyn 3700 analyzer, as previously reported [38].

For qPCR assays, Tregs and CD4^+^Tconvs were sorted from spleen of unmanipulated B10.D2 mice using FACS Aria III (Becton Dickinson).

#### Methylation-sensitive restriction enzyme and qPCR (MSRE-qPCR)

Methyl-sensitive PCR were used to analyze the methylation status of the CpG island of the *Foxp3* enhancer and the *IL-2* promoter, as previously described [29, 39, 40]. Genomic DNA was prepared using PureLink^TM^ Genomic DNA Mini Kit (Invitrogen, CA, USA) and quantified. One hundred and fifty nanograms of genomic DNA isolated from murine splenocytes was digested overnight at 37 °C with 10U of HpaII or MspI enzymes for *Foxp3* enhancer and with 10U of PleI or MlyI enzymes for *IL-2* promoter analyses. The CpG islands and *β*-act genes were PCR amplified using the following specific primers: *Foxp3* enhancer CpG island, 5′-ATCCTCGCCATCGTCTTCCTCAT-3′ (forward) and 5′-CCTGTTCTGGCTTTCTCATTGGCT-3′ (reverse); *IL-2* CpG island, 5′- CTGTTTCCATGCTGAAGGTC-3′(forward) and 5′-GGGTGATGCTCCAACTTCAT-3′ (reverse); *β-act* (GenBank accession no. U89400), 5′-TAGCACCATGAAGATCAAG-3′ (forward) and 5′-CCTGCTTGCTGATCCACAT-3′ (reverse). Specific primers designed on CpG islands were selected according to the CpG island methylation tool server (http://urogene.org/methprimer). Quantitative real-time PCR was performed using the LightCycler 480 thermocycler and the KAPA SYBR FAST qPCR kit, following the manufacturer’s instructions (Kapa Biosystems, Wilmington, USA,), subsequently the melting curve analysis was performed. Data were normalized using the *β*-act Endogenous Control and *β*-act specific primer for uncut region by HpaII, MspI, PleI and MlyI. Differences between males and females were taken into account for *Foxp3* analyses, since this gene gene is located on the X chromosome.

### TCR Vβ CDR3-size spectratype analysis

The TCR Vβ CDR3-size spectratyping was performed as previously reported [35] (see supplemental data for more details).

### Statistical analyses

The Mann-Whitney *U* test was used to compare data between experimental groups. Results are expressed as mean with SEM (for scl-GVHD scores), or median, 25th and 75th percentiles of the distribution (boxes), and whiskers extending to the 5th and 95th percentiles. The analysis of quantitative real time PCR data was based on 2^−ΔΔCT^ values. Two-tailed *P* values <0.05 were considered as statistically significant. Statistical analyses were performed with the GraphPad Prism 5.0 software.

## Results

### AZA mitigates scl-cGVHD

**Fig. 1:**
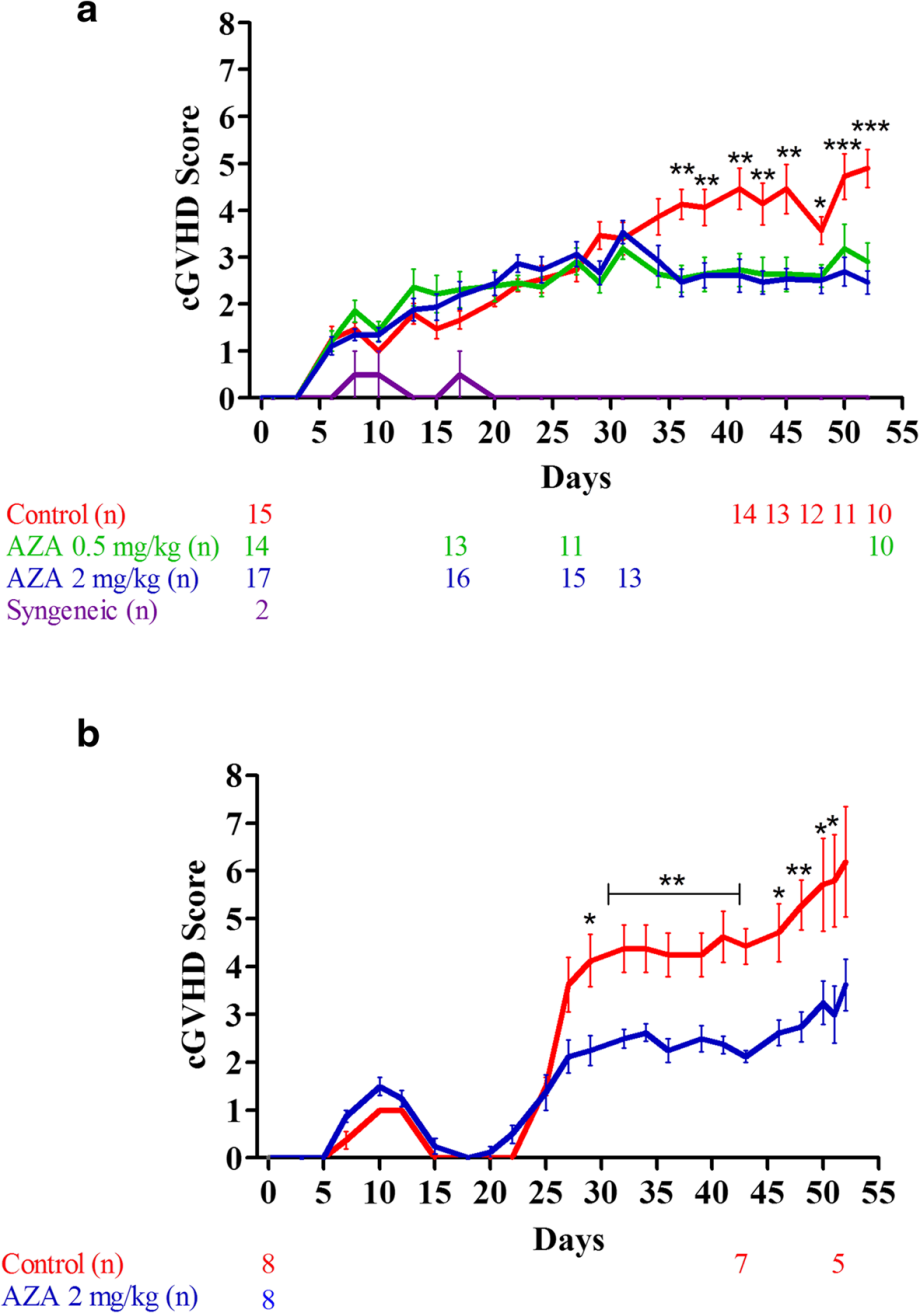
Azacytidine mitigates scl-cGVHD. Balb/cJ mice were injected i.v. with 10.10^6^ bone marrow cells and 70.10^6^ splenocytes from B10.D2 donor mice (or from Balb/cJ mice in case of syngeneic transplantation) after lethal irradiation. Mice were then given (or not) azacytidine (AZA) administered subcutaneously every 48 h from day +10 to day +30. Animals were individually scored three times/week and animals reaching a score of 8/10 were sacrificed (evolutions of the number of animals along the course of each experiment are shown under each graph). **a** Pooled GVHD scoring of two independent groups (two distinct experiments) of mice given or not AZA (0.5 or 2 mg/kg) showing lower GVHD scores in AZA-treated mice. Two out of 15 control mice died during the experiments due to achievement of the critical score of 8/10 and three others because of weight loss >20 %. Concerning mice receiving AZA 0,5 mg/kg, three out of 14 mice were sacrificed during the experiment because a weight loss >20 % and 1 due to achievement of the critical score. For mice treated with AZA 2 mg/kg, two mice were sacrificed because of a weight loss >20 % and two did not survive the anesthesia administered for skin biopsy performed during the experiment (day +29). **b** GVHD scoring of a third cohort of mice given or not AZA (2 mg/kg only) confirming lower GVHD scores in treated mice. Three out of eight control mice were sacrificed because they achieved the critical score of 8/10, while all AZA mice survived without achieving the critical GVHD score. Results are expressed as mean with SEM. *P < 0.05; **P < 0.01; ***P < 0.001

In a first set of two consecutive experiments, Balb/cJ mice were injected i.v. with 10.10^6^ bone marrow cells and 70.10^6^ splenocytes from B10.D2 donor mice after lethal irradiation. Mice were then given (or not) azacytidine (AZA) administered subcutaneously every 48 h from day +10 to day +30. We observed that AZA, at either 0.5 or 2 mg/kg doses, significantly mitigated cGVHD with mice from the AZA group having significantly lower cGVHD scores than control mice from day +35 to the end of the experiment (day +52) (Fig. 1a). In accordance with the similar cGVHD scores between the two groups of mice on day +29 after transplantation, skin fibrosis (assessed by trichrome staining) was similar in AZA and control mice at that time point (data not shown).

**Fig. 2:**
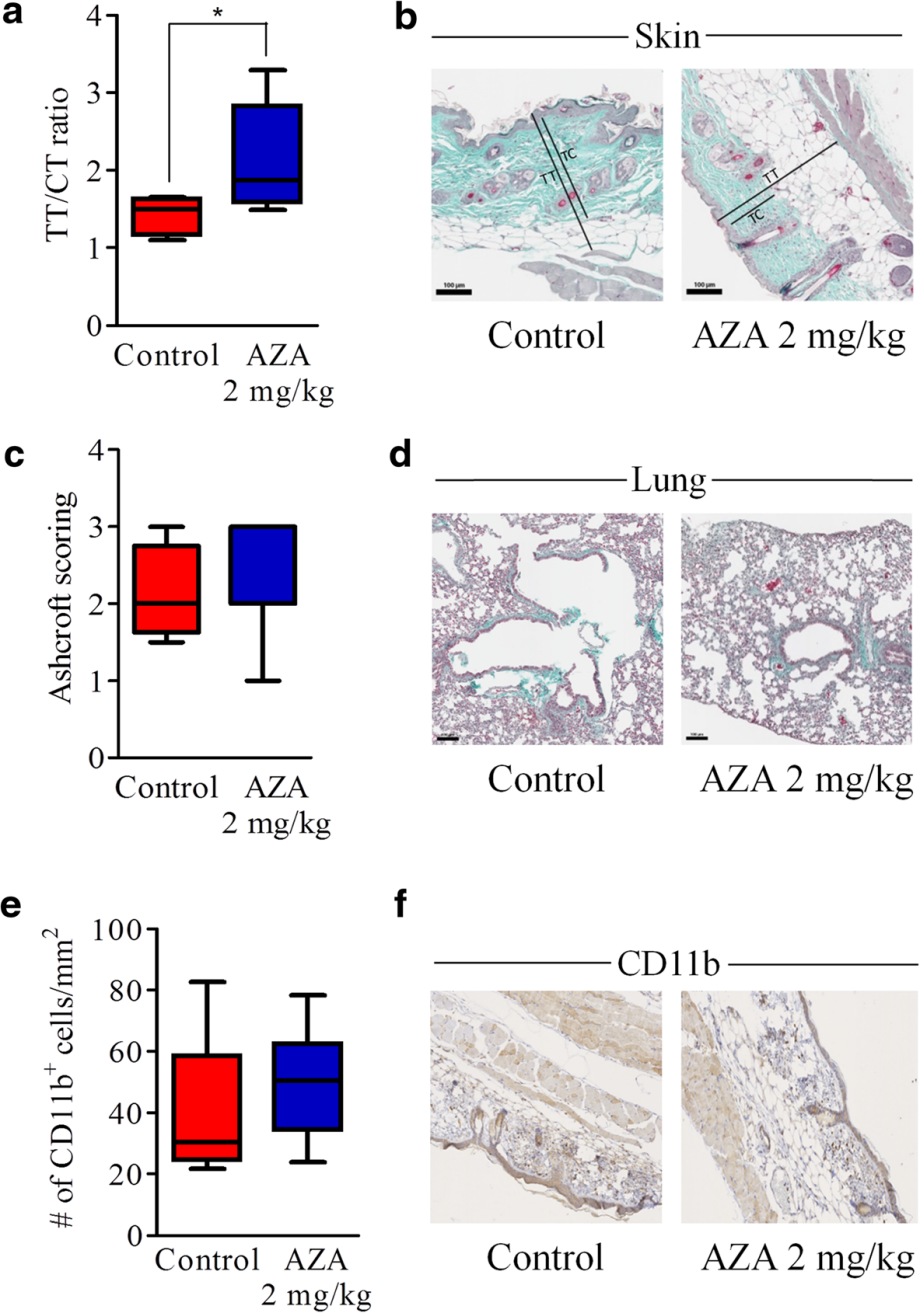
Azacytidine mitigates skin fibrosis on day +52. Balb/cJ mice were injected i.v. with 10.10^6^ bone marrow cells and 70.10^6^ splenocytes from B10.D2 donor mice after lethal irradiation. Mice were then given (or not) azacytidine (AZA, 2 mg/kg) administered subcutaneously every 48 h from day +10 to day +30. At sacrifice (day +52), histology was performed on lung and skin samples to evaluate fibrosis (Goldner’s Trichrome) and monocyte infiltration (CD11b staining). **a**, **b** Quantification (**a**) and representative images (**b**) (Trichrome 150×) of skin fibrosis in control (*n* = 5) and AZA-treated (*n* = 7) mice. Ratios were calculated by dividing total thickness (TT, from epidermis to sub-cutaneous muscle layer) by collagen thickness (CT, stained by Goldner’s Trichrome). **c**, **d** Ashcroft [36] quantification (**c**) and representative images (**d**) (Trichrome 100×) of lung fibrosis in control (*n* = 4) and AZA-treated (*n* = 7) mice. **e**, **f** Quantification (**e**) and representative images (**f**) (IHC 100×) of monocyte infiltration in the skin of control (*n* = 5) and AZA-treated (*n* = 8) mice. Results are expressed as median, 25th and 75th percentiles of the distribution (boxes), and whiskers extending to the 5th and 95th percentiles. *P < 0.05

In a third experiment, we confirmed that AZA given at 2 mg/kg decreased cGVHD (Fig. 1b) and observed that decitabine (DAC, 0.75 mg/kg, *n* = 5) also mitigated cGVHD (Additional File 1: figure S1). We further evaluated skin fibrosis in the mice at the end of the experiment. As shown in Fig. 2, AZA-treated mice had higher total thickness (from epidermis to sub-cutaneous muscle layer)/collagen thickness (TT/CT) ratios, highlighting less skin fibrosis (Fig. 2a, b), but similar (and mild) lung fibrosis (Fig. 2c, d) than control mice on day 52 after transplantation. Interestingly, infiltration by monocytes/macrophages was similar in AZA and control mice on that day (Fig. 2e, f).

In a fourth experiment, we investigated whether AZA administered every 4 days from days 10 to 30 also prevented cGVHD. As shown in Additional File 1: figure S1, this was not the case, with AZA and control mice exhibiting similar cGVHD scores throughout the experiment.

### Impact of AZA on blood cell counts and immune recovery

**Fig. 3:**
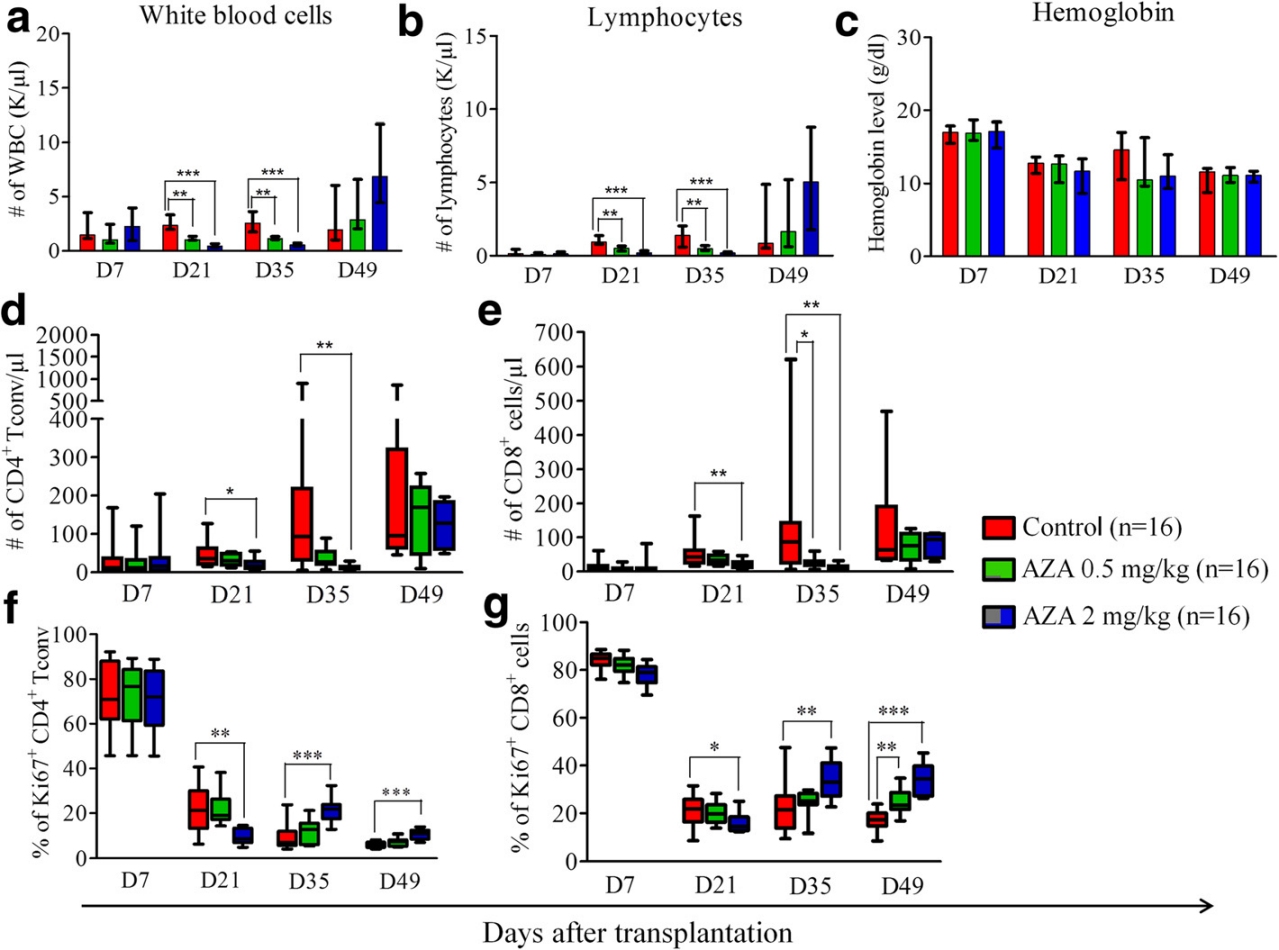
Impact of azacytidine on blood cell counts and immune recovery. Balb/cJ mice were injected i.v. with 10.10^6^ bone marrow cells and 70.10^6^ splenocytes from B10.D2 donor mice after lethal irradiation. Mice were then given (or not) azacytidine (AZA, 0.5 mg/kg or 2 mg/kg), administered subcutaneously every 48 h from day +10 to day +30. **a**–**c** Impact of AZA on total white blood cell counts (**a**), total lymphocyte counts (**b**) and hemoglobin levels (**c**). **d**–**g** FACS analyses performed on mice blood at various time points showing lower counts and proliferation of CD4^+^Tconvs (**d**, **f**) and of CD8^+^ T cells (**e**, **g**) during AZA administration but higher CD4^+^Tconvs (F) and of CD8^+^ T cells proliferation after AZA discontinuation. Results are expressed as median, 25th and 75th percentiles of the distribution (boxes), and whiskers extending to the 5th and 95th percentiles. *P < 0.05; **P < 0.01; ***P < 0.001

We next assessed the impact of AZA on blood cell counts and immune recovery in the first two experiments. We observed that AZA significantly decreased total white blood cell (Fig. 3a) and lymphocyte (Fig. 3b) counts on days +21 and +35 after transplantation, while hemoglobin levels remained comparable in both groups (Fig. 3c). Further, AZA significantly decreased absolute counts of both CD4^+^Tconvs (Fig. 3d) and CD8^+^ T cells (Fig. 3e) from day +21 to day +35 after transplantation. In contrast, there was a trend for higher absolute lymphocyte counts in AZA-treated mice on day +52. This was due to significantly higher numbers of CD3^-^ lymphocytes (probably mainly B cells) in AZA-treated mice (*P* = 0.01), as observed previously by Choi et al. in a mouse-to-mouse model of acute GVHD [31]. This is probably an indirect phenomenon (better B lymphopoiesis due to less intense cGVHD in AZA-treated mice) since this was not observed in mice given syngeneic transplantation (Additional File 1: figure S2).

Interestingly, the proportion of proliferative CD4^+^Tconvs and CD8^+^ T cells (as assessed by Ki67 expression) was lower in AZA-treated mice on day +21 after transplantation (Fig. 3f, g) (this effect was dose dependent since it was only observed in mice given 2 mg/kg of AZA), suggesting an anti-proliferating effect of AZA on T cells. However, upon AZA discontinuation, CD4^+^Tconvs and CD8^+^ T cells proliferation was restored and higher numbers of Ki67+ cells were observed in AZA treated mice at days +35 and +49 post-transplant. Comparable qualitative observations were made on day 35 with DAC in the third experiment (Additional File 1: figure S3).

### AZA increases Treg frequency and induces demethylation of Foxp3 enhancer

**Fig. 4:**
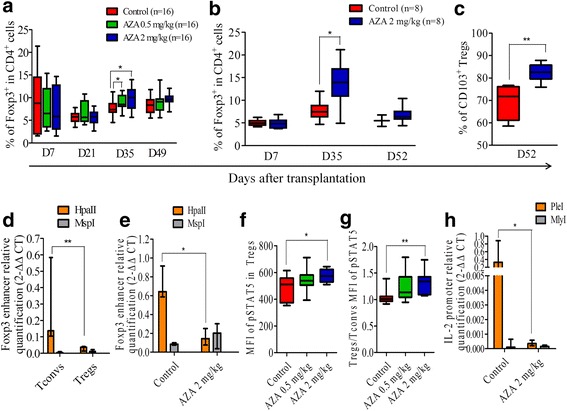
Azacytidine increases Treg frequency and induces demethylation of *Foxp3* enhancer and *IL-2* promoter. Balb/cJ mice were injected i.v. with 10.10^6^ bone marrow cells and 70.10^6^ splenocytes from B10.D2 donor mice after lethal irradiation. Mice were then given (or not) azacytidine (AZA, 0.5 mg/kg or 2 mg/kg) administered subcutaneously every 48 h from day +10 to day +30. **a**, **b** FACS analyses showing the Tregs frequency in two cohorts of mice combined (**a**) and in a third cohort of mice (**b**). **c** FACS analysis of activated CD103^+^ cells among Tregs in the spleens of AZA on day +52 (data from the third cohort). **d**, **e** Methylation status of the *Foxp3* enhancer assessed by methylation-sensitive restriction enzyme and qPCR performed on genomic DNA of Tregs (*n* = 3) and Tconvs (*n* = 9) sorted from the spleen of three unmanipulated B10.D2 mice (**d**) or of the spleens of control (*n* = 4) or AZA-treated (*n* = 5) recipient Balb/cJ mice at day +52 post transplantation (**e**). **f** FACS comparison of phosphorylated-STAT5 mean fluorescence intensity (MFI) between Tregs in spleen of control (*n* = 8) and AZA-treated (*n* = 9) mice. **g** Comparison of ratio, for each mouse treated or not with AZA, of pSTAT5 MFI of Tregs *versus* pSTAT5 MFI of Tconvs. **h** Methylation status of the IL-2 promoter assessed by MSRE-qPCR performed on genomic DNA of spleens from control (*n* = 4) and AZA-treated (*n* = 5) recipient Balb/cJ mice at day +52 post transplantation. Results are expressed as median, 25th and 75th percentiles of the distribution (boxes), and whiskers extending to the 5th and 95th percentiles. *P < 0.05; **P < 0.01

Given that AZA induces Tregs in vitro, we assessed the impact of HMA on Treg reconstitution. In the two first experiments, we observed that AZA increased the frequency of Tregs (Fig. 4a) and decreased the CD4^+^Tconv/Treg ratio (*P* = 0.046 for mice treated with AZA 0.5 mg/Kg and *P* = 0.029 for mice treated with AZA 2 mg/kg) on day +35 after transplantation. In contrast, absolute counts of Tregs were significantly lower in AZA-treated mice on days +21 and +35 after transplantation (data not shown). Comparable findings were observed in the third experiment with AZA 2 mg/kg (Fig. 4b) and DAC (Additional File 1: figure S3). Further, interestingly, Tregs from the spleens of AZA- and DAC-treated mice expressed more frequently the activation marker CD103 than those from control mice on day +52 after transplantation, suggesting higher efficacy (Fig. 4c and Additional File 1: figure S3). A similar impact of AZA on Tregs was observed in mice given syngeneic grafts (Additional File 1: figure S4), demonstrating that the impact of AZA on Treg reconstitution was independent of cGVHD.

Previous studies have reported that CD8^+^Foxp3^+^ regulatory T cells expanded after experimental allogeneic bone marrow transplantation in mice and mitigated experimental acute GVHD [41, 42]. This led us to look whether AZA promoted the generation of CD8^+^Foxp3^+^ regulatory T cells in vivo. However, we failed to observe significant numbers of CD8^+^Foxp3^+^ regulatory T cells in control and in AZA-treated mice after either allogeneic or syngeneic transplantation (Additional File 1: figure S5).

The higher frequency of Tregs in AZA-treated mice on day +35 prompted us to assess the impact of AZA on the methylation status of the *Foxp3* enhancer. We first quantified the methylation status of *Foxp3* enhancer in CD4^+^ Tconvs and Tregs isolated from the spleen of healthy mice. For this, DNA was extracted and digested with two restriction enzymes, Hpall and Mspl, both recognizing CG sequences. It is worth to note, however, that Hpall is not able to cut methylated sequences. As expected, no differences were observed between groups after Mspl digestion, while Hpall digestion demonstrated reduced methylation levels of the *Foxp3* enhancer in Tregs in comparison with CD4^+^Tconvs, in agreement with studies previously published by others [29, 39] (Fig. 4d). We then assessed the methylation status of the *Foxp3* enhancer in total spleen cells collected in cGVHD mice at the end of the experiment (on day +52 after transplantation). As hypothesized, we observed significantly less methylation of the *Foxp3* enhancer in AZA-treated mice (Fig. 4e). Similar findings were made in DAC-treated mice (Additional File 1: figure S3).

Since the mTOR pathway is constitutively inactivated in Tregs, they rely mainly on the STAT5 pathway for IL-2 signaling [37, 43]. In order to further investigate the impact of AZA on day 30 after transplantation, we studied a fifth cohort of mice given or not AZA 0.5 or 2 mg/kg every other day from day +10 to day +30. In that cohort, mice were sacrificed on day +30 after transplantation. We observed that Tregs recovered from the spleen of mice treated with AZA 2 mg/kg had higher levels of phosphorylated STAT5 (pSTAT5) than Tregs from control mice (Fig. 4f). Further, they had also higher Tregs versus Tconvs ratio of STAT5 phosphorylation level than control animals (Fig. 4g). This higher IL-2 signaling in Tregs from AZA-treated mice than in those from control mice prompted us to assess the methylation status of the IL-2 promoter in spleen cells from control and AZA-treated mice on day +52 after transplantation. For this, DNA was digested with 2 restriction enzymes, PleI and MlyI. Both enzymes recognize same CG sequences but PleI is sensitive to methylation and is not able to cut when these sequences are methylated. No differences were observed between groups after MlyI treatment used as control while PleI digestion demonstrated reduced methylation levels in AZA-treated compared to control mice (Fig. 4h), in agreement with findings previously published by other groups [44–46].

### AZA increases Treg frequency in thymus on day +35

**Fig. 5:**
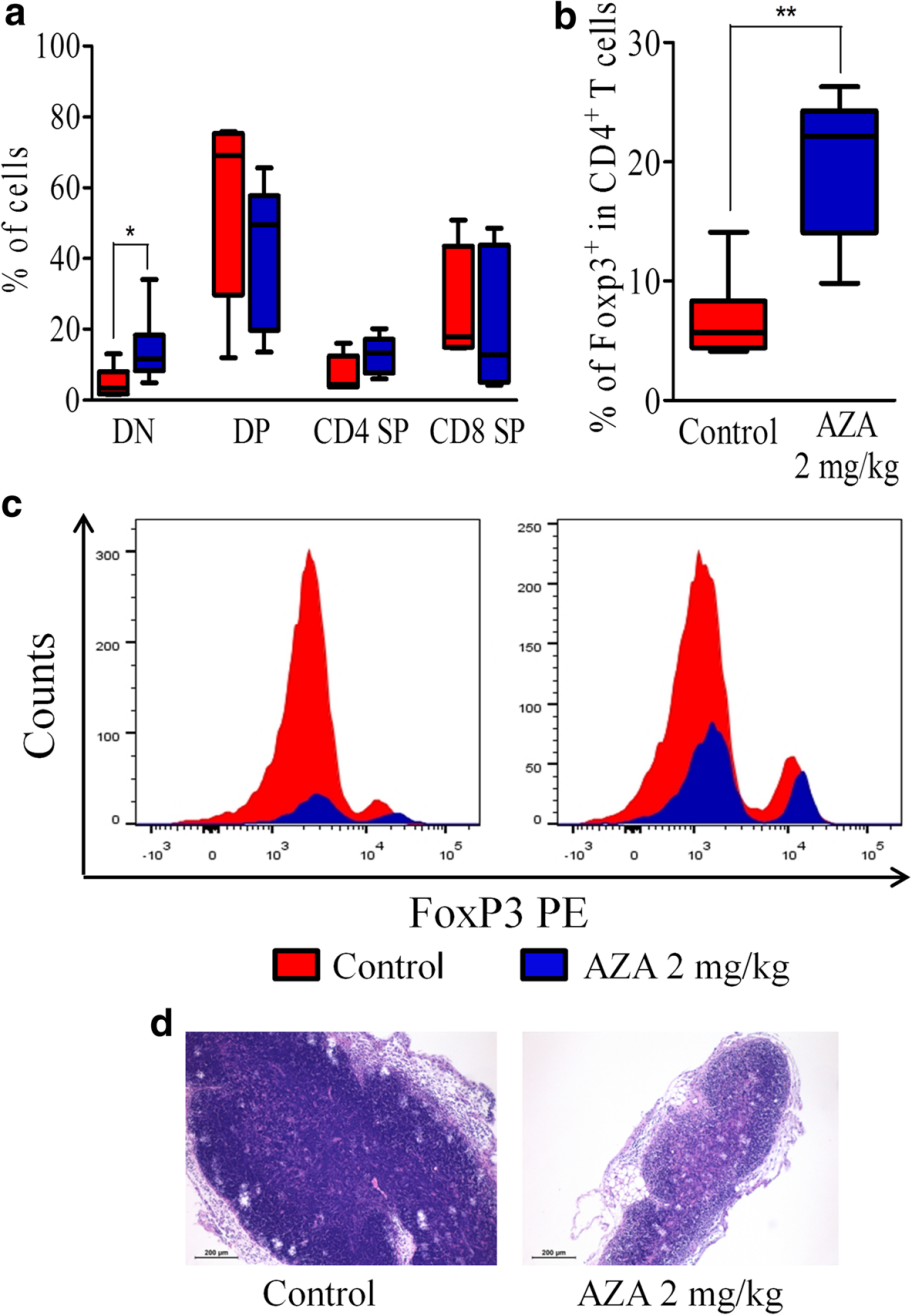
Azacytidine increases Treg frequency in the thymus on day +35. Balb/cJ mice were injected i.v. with 10.10^6^ bone marrow cells and 70.10^6^ splenocytes from B10.D2 donor mice after lethal irradiation. Mice were then given (or not) azacytidine (AZA, 2 mg/kg) administered subcutaneously every 48 h from day +10 to day +30. On day +35, thymus from control (*n* = 6) and AZA-treated mice (*n* = 6) were collected. **a**, **b** FACS analyses showing the frequencies of double negative CD4/CD8 cells (DN), double-positive CD4/CD8 cells (DP), single-positive CD4 cells (CD4 SP), and single-positive CD8 cells (CD8 SP) populations in the thymus (**a**) and the frequency of regulatory T cells among CD4 single-positive T cells (**b**). **c** Representative graphs showing CD4^+^ T cells discriminated into conventional (CD4^+^Foxp3^−^) and regulatory T cells (CD4^+^Foxp3^+^) in control and AZA-treated mice. **d** Histological evaluation (Hematoxylin-eosin, 100×) of thymic architecture in representative control and AZA-treated mice. Results are expressed as median, 25th and 75th percentiles of the distribution (boxes), and whiskers extending to the 5th and 95th percentiles. *P < 0.05; **P < 0.01

Given that the thymus is the site of natural Treg generation on the one hand, and that thymic defects have been involved in the pathogenesis of cGVHD on the other hand [47, 48], we compared the thymus of AZA-treated and control mice on day +35 after transplantation in a last experiment. We observed that AZA-treated mice had less cellularity, higher percentages of double negative thymocytes, and a trend for lower percentages of double positive lymphocytes (Fig. 5a). Interestingly, there was a significantly higher proportion of Tregs (defined as CD90.2^+^CD4^+^CD8^-^Foxp3^+^) in the thymus of AZA-treated mice than in those from control mice (Fig. 5b and c). However, the proportion of Foxp3 double positive thymocytes was very low and similar in both groups of mice (data not shown), suggesting that the higher Treg frequency observed in the thymus of AZA-treated mice was due to higher Treg thymic recirculation. Finally, although adequate cortico-medullar differentiation was conserved in both control and AZA-treated mice, thymus from AZA-treated mice tended to have smaller sizes (Fig. 5d), in agreement with their lower cellularity (Fig. 5c).

### AZA does not restrict T-cell receptor Vβ (TCRVβ) repertoire

Given the anti-proliferative impact of AZA on T cells, we compared the diversity of the TCRVβ repertoire on days +35 and +52 after transplantation. As shown in Additional File 1: figure S6, AZA did not significantly impact TCRVβ repertoire diversity.

## Discussion

Previous studies have demonstrated that HMA induced the generation of Tregs in vitro, and prevented acute GVHD in mouse-to-mouse models of transplantation [30, 31]. Given the important divergences in the pathogenesis of acute and chronic GVHD, we sought to investigate the impact of AZA in a well-established CD4-dependent murine model of scl-cGVHD. We selected the dose and schedule of AZA administration based on previous publications in mouse-to-mouse models of acute GVHD [30, 31], and on prior observations by our group in a humanized mouse model of GVHD [49]. The dose of 2 mg/kg is close to the dose used in patients with acute myeloid leukemia (75 mg/m^2^) [50, 51]. Several observations were made.

First and most importantly, AZA indeed mitigated cGVHD. This achievement was dependent on the schedule of AZA administration since injection of AZA 2 mg/kg every 2 days but not every 4 days was able to decrease cGVHD severity. The ability of AZA to prevent cGVHD in our mouse model is in line with the low incidence of cGVHD observed in patients receiving donor lymphocytes infusion and AZA as treatment of acute myeloid leukemia relapse after allo-HCT [52], and consistent with the very low incidence of extensive cGVHD observed in patients given prophylactic AZA after allo-HCT [53, 54], although the latter patients also received alemtuzumab in the conditioning regimen (which is associated with a very low incidence of cGVHD by itself [55]).

At least two mechanisms were involved in cGVHD prevention by AZA. First, AZA had an antiproliferative effect on Tconvs during treatment. This is in line with previous reports that demonstrated an anti T-cell proliferative impact of HMA in vitro [30, 31]. Secondly, mice given AZA had significantly higher Treg frequencies on day 35 after transplantation, while a higher frequency of Tregs had an activated (CD103^+^) phenotype on day +52 after transplantation. These observations are particularly relevant given that a prior study had observed that in vivo-activated CD103^+^ donor Tregs were much more efficient than resting donor Tregs at ameliorating ongoing cGVHD [26]. These results are in concordance with the higher Treg levels observed in patients given prophylactic AZA after allo-HCT [54].

Interestingly, we also observed a higher frequency of Tregs in the thymus of AZA-treated mice on day +35 after transplantation. Whether this was due to a higher neogeneration of Tregs by the thymus or to a higher frequency of peripheral Tregs recirculating into the thymus remains to be determined in further experiment using Rag2pGFP mice [56]. Indeed, we did not observe any increase in the proportion of double positive thymocytes expressing Foxp3 in the thymus of AZA-treated mice compared to those of control animals.

It is likely that the higher Treg frequency observed in AZA-treated mice on day +35 is the result of demethylation of several genes. This assumption is supported by the demonstration of lower methylation status of both the *Foxp3* enhancer and the *IL-2* promoter in the spleens from AZA-treated mice in our study. Demethylation of the *IL-2* promoter is particularly relevant, given the primordial role of IL-2 in Treg homeostasis [20]. In line with the demethylation status of the *IL-2* promoter in AZA-treated mice, we observed higher pSTAT5 levels in Tregs from AZA-treated than from control mice on day +30 after transplantation, confirming that IL-2 signaling in Tregs was higher in AZA-treated than in control mice.

Finally, as expected, AZA decreased blood counts and delayed immune recovery. However, this negative impact of the drug was rapidly corrected after AZA discontinuation. Further, interestingly, AZA did not significantly reduce the diversity of the TCRVβ repertoire.

## Conclusions

In summary, our results indicate that AZA prevented scl-cGVHD in a well-established murine model of cGVHD. This was correlated with the demonstration of anti-proliferative effects of AZA on T cells, as well as with a higher frequency of Tregs (and particularly of activated CD103^+^ Tregs) in mice receiving AZA. These observations might serve as basis for pilot studies of AZA administration for cGVHD prevention in patients at high risks for cGVHD or for treatment of steroid-refractory scl-cGVHD.

## Abbreviations

allo-HCT, allogeneic hematopoietic cell transplantation; AZA, azacytidine; cGVHD, chronic graft-versus-host disease; DAC, decitabine; Foxp3, forkhead box protein 3 factor; HMA, hypomethylating agents; Scl-cGVHD, sclerodermatous cGVHD; Tconvs, conventional CD4^+^ T cells; TCRVβ, T-cell receptor *Vβ*; Tregs, regulatory T cells

## Additional file

Additional file 1: Figure S1. A): Azacytidine administered every fourth day failed to prevent cGVHD. (B) Decitabine administered every other day ameliorated cGVHD. **Figure S2**: Impact of Azacytidine on blood counts and immune recovery after syngeneic transplantation. **Figure S3**: Impact of decitabine on skin, lungs and immune recovery. **Figure S4**: Azacytidine enhances Treg frequency and functions after syngeneic transplantation. **Figure S5**: Azacytidine does not induce the generation of CD8^+^FoxP3^+^ regulatory T cells after allogeneic or syngeneic transplantation. **Figure S6**: Azacytidine does not restrict T-cell Vβ (TCR Vβ) repertoire diversity on day +35 and +52. (DOCX 10679 kb)
