# Supplementary material for: Azacytidine mitigates experimental sclerodermic chronic graft-versus-host disease
Source: J Hematol Oncol. 2016 Jul 4;9:53. doi: 10.1186/s13045-016-0281-2 (PMC4932697; doi:10.1186/s13045-016-0281-2)

**ADDITIONAL METHODS**

***TCR Vβ CDR3-size spectratype analysis***

At sacrifice (days +35 in the fifth experiment or day +52 in the third experiment), a piece of spleen was conserved in RNA Later stabilization reagent (Qiagen, Venlo, The Netherlands). RNA was then extracted using the RNEasy mini kit (Qiagen) following the manufacturer’s instructions. Genomic DNA was removed using recombinant RNase-free DNaseI (Roche, Vilvoorde, Belgium). cDNA was synthesized from RNA (2 µg) using oligo(dT)_18_ primers with the “Transcriptor First Strand cDNA synthesis kit” (Roche). Semi-nested PCR was performed using sense primers for a panel of murine Vβ families and two Cβ anti-sense primers, the second being fluorescently labelled (IDT Technologies, Leuven, Belgium). All PCR reagents were purchased from Applied Biosystems (Life Technologies, Gent, Belgium). The fluorescently labelled PCR products were run together with GeneScan ROX 500 Size Standard (Applied Biosystems) on a “DNA Analyzer 3730“ (Applied Biosystems) 48-capillary electrophoresis system at the GIGA-Research Genomics facility of the University of Liège. CDR3-size spectratype analysis was performed with GeneMapper version 4.0 Software (Applied Biosystems).

**SUPPLEMENTAL FIGURES**

**Supplemental Figure 1. A) Azacytidine administered every fourth day failed to prevent cGVHD.** Balb/cJ mice were injected i.v. with 10.10^6^ bone marrow cells and 70.10^6^ splenocytes from B10.D2 donor mice after lethal irradiation. Mice were then given (or not) azacytidine (AZA), administered subcutaneously every 4 days from day +10 to day +30. Animals were individually scored 3 times/week and nimals reaching a score of 8/10 were sacrificed to avoid unnecessary pain according to our local ethics committee guidelines. Loss of control and AZA-treated (0.5 mg/kg) mice during the experiment was due to achievement of the critical score of 8/10. The only mice lost during the experiment in the group receiving AZA 2 mg/kg was due to a weight loss >20%. **(B) Decitabine administered every other day ameliorated cGVHD.** Mice were transplanted as described above and decitabine (DAC, 0.75 mg/kg) was injected (or not) subcutaneously every 48h from day +10 to day +30. The figure shows lower GVHD scores in DAC-treated than in control mice. All mice lost during this experiment were due to achievement of the critical score of 8/10.


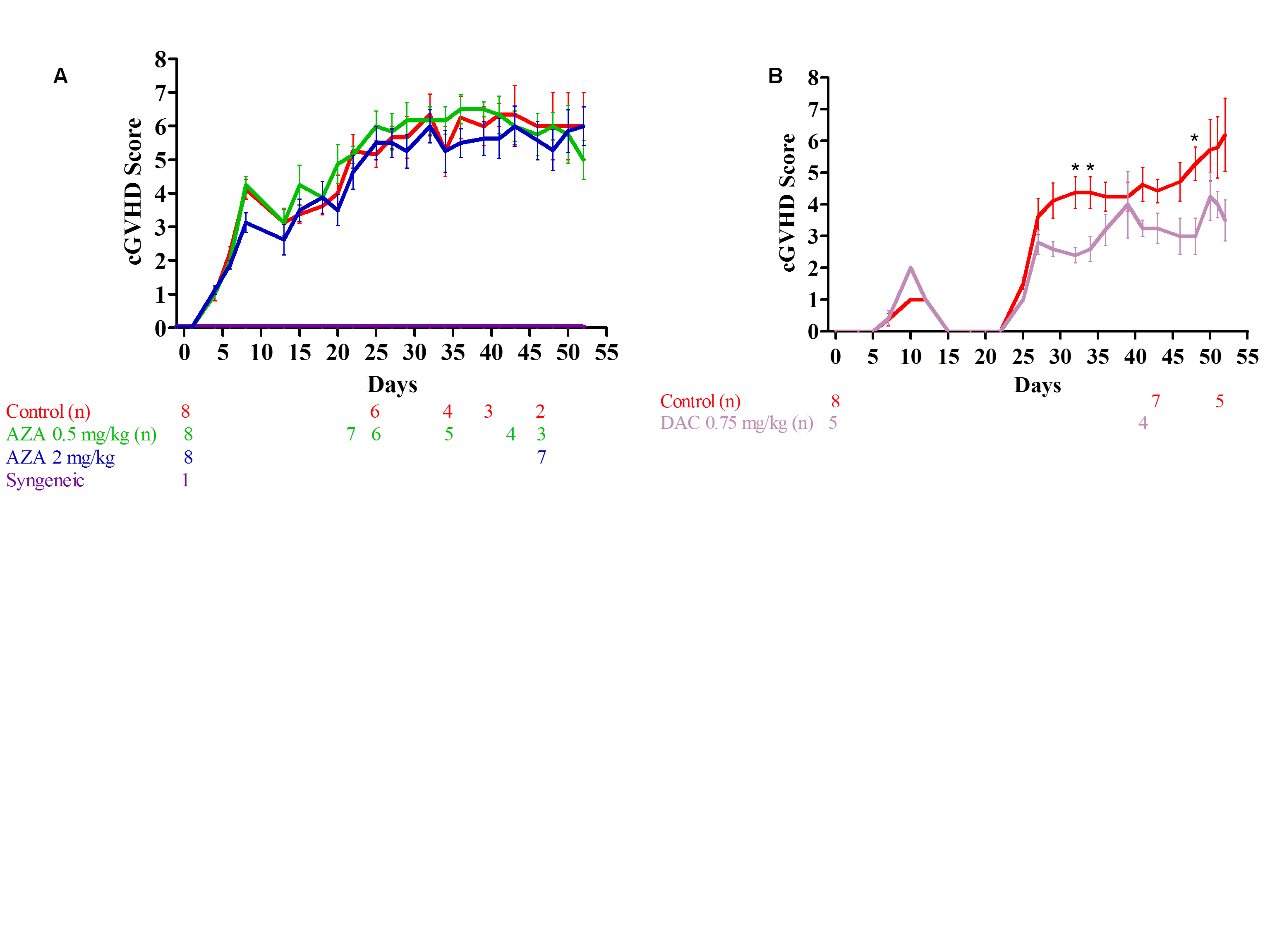


**Supplemental Figure 2. Impact of azacytidine on blood counts and immune recovery after syngeneic transplantation**

Balb/cJ mice were injected i.v. with 10.10^6^ bone marrow cells and 70.10^6^ splenocytes from Balb/cJ donor mice after lethal irradiation. Syngeneic mice were then given (or not) azacytidine (AZA, 0.5 mg/kg or 2 mg/kg), administered subcutaneously every 48h from day +10 to day +30. (A-B-C) Impact of AZA on hemoglobin levels and blood counts (as determined by a Cell-Dyn 3700 analyser). (D-E-F-G) FACS analyses performed on blood at various time points showing lower T-cell numbers during and directly after the end of AZA administration and higher T-cell proliferation after AZA withdrawal. * = *P* < 0.05.

**
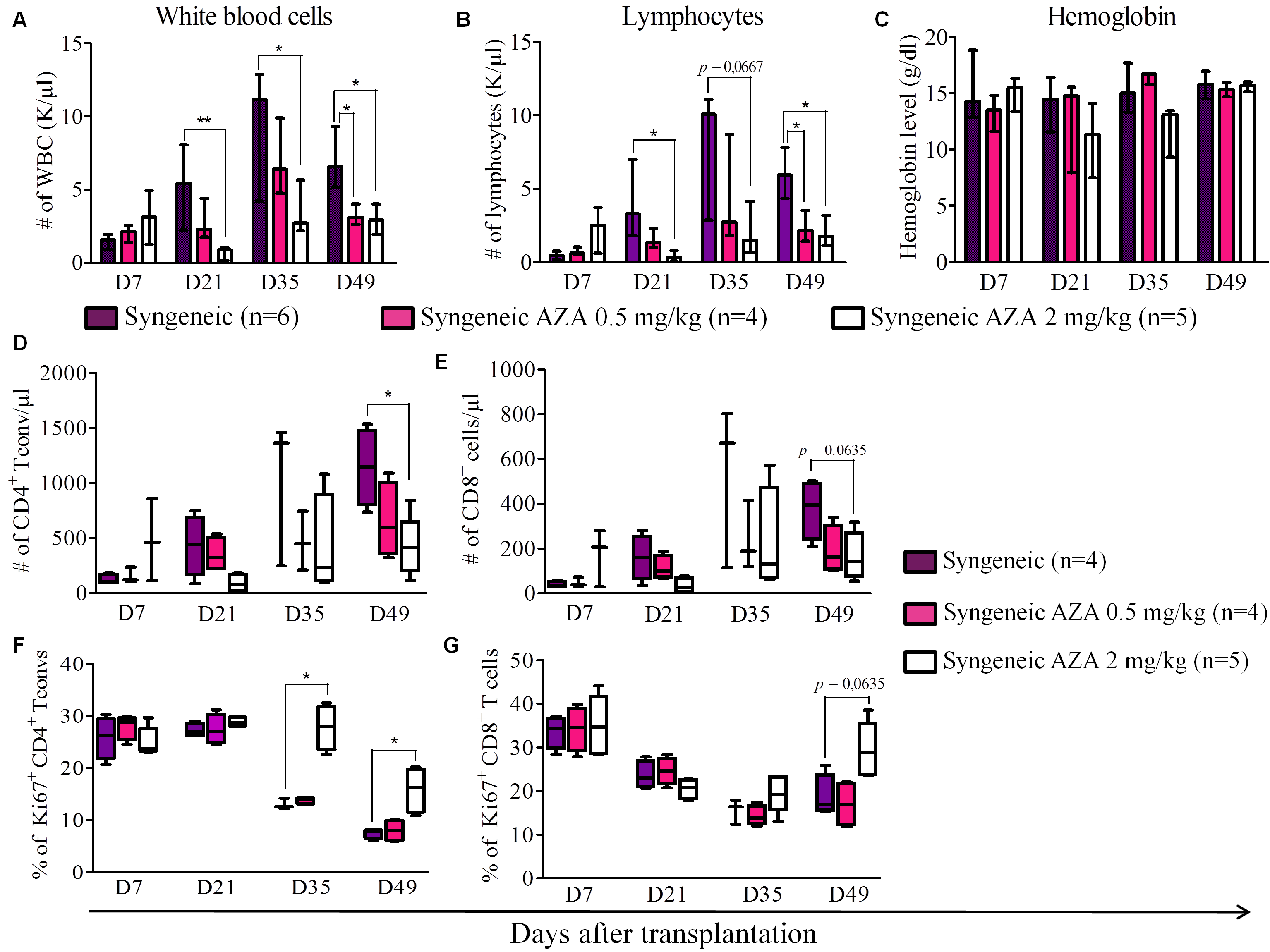
**

**Supplemental figure 3. Impact of decitabine on skin, lungs and immune recovery.**

Balb/cJ mice were injected i.v. with 10.10^6^ bone marrow cells and 70.10^6^ splenocytes from B10.D2 donor mice after lethal irradiation. Mice were then given (or not) decitabine (DAC, 0.75 mg/kg), administered subcutaneously every 48h from day +10 to day +30. At sacrifice (day +52), lung and skin samples from the upper back from each group of mice were harvested and fixed in 10% formaldehyde before being paraffin-embedded. Skin and lung sections were then stained with Masson’s Trichrome to quantify fibrosis while CD11b was stained in skin sections to quantify monocyte/macrophage infilitration. (A) Histological evaluation of skin fibrosis. Ratios were calculated by dividing total thickness (from epidermis to sub-cutaneous muscle layer) by collagen thickness (stained by Masson’s trichrome). There was a suggestion for higher ratios (i.e. more fibrosis) in control (n=5) than in DAC-treated (n=3) mice, although the difference was not statistically significant perhaps due to the low number of DAC mice. (B) Histological evaluation of lung fibrosis. Stained lung sections were quantified using the Ashcroft scoring system. Mild fibrosis was observed in the lungs of control mice (n=4) and was DAC (n=4). (C) Histological evaluation of monocyte/macrophage infiltration in the skin after CD11b staining. Absolute counts of CD11b+ cells were quantified in three different hotspots for each slide. The total number of positive cells was normalized to the number of CD11b^+^ cells per mm^2^ for each sample. No differences were observed between control (n=5) and DAC-treated mice (n=4). (D-E-F) FACS analyses performed on blood at various time points showing higher T-cell proliferation after DAC withdrawal. (G) FACS analyses showing the Treg frequency in mice treated or not with DAC, showing a higher frequency of Tregs on day 35 in DAC-treated mice. (H) Demonstration of an increased frequency of activated Tregs (CD103^+^) in the spleens of DAC-treated mice on day +52. (I) Methylation status of the *Foxp3* enhancer. Genomic DNA was collected from the spleen of recipient Balb/cJ mice at day 52 post transplantation. Then, DNA was digested with 2 restrictions enzymes, Hpall and Mspl. Both enzymes recognize CG sequences but Hpall is not able to cut when these sequences are methylated. Each sample was performed in duplicate. Female and male subjects were separately analyzed since the *Foxp3* gene is located on X-chromosomes. As expected, no differences were observed between groups after Mspl digestion, while Hpall digestion demonstrated reduced methylation levels of the *Foxp3* enhancer in DAC-treated mice (n=5) compared to controls (n=4). (J) Methylation status of the IL-2 promoter. Genomic DNA was collected from spleens of recipient Balb/cJ mice at day +52 post transplantation. DNA was digested with 2 restrictions enzymes, PleI and MlyI. Both enzymes recognize the same CG sequences but PleI is sensitive to methylation and so not able to cut when theses sequences are methylated. No differences were observed between DAC-treated mice (n=5) and controls (n=4) after PleI or MlyI treatment (used as control). * = *P* < 0.05; ** = *P* < 0,01.


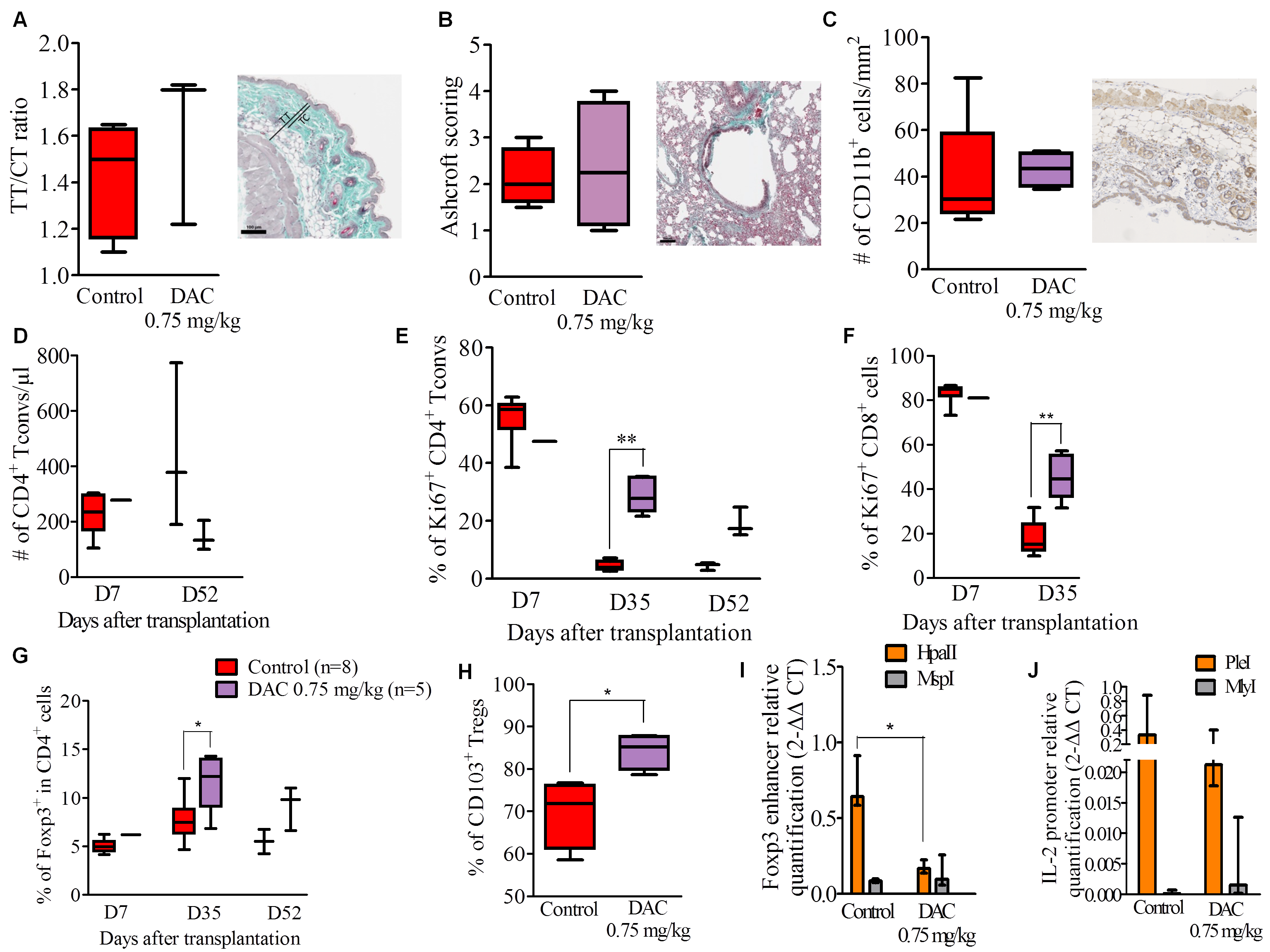


**Supplemental Figure 4. Azacytidine enhances Treg frequency and functions after syngeneic transplantation**

Balb/cJ mice were injected i.v. with 10.10^6^ bone marrow cells and 70.10^6^ splenocytes from Balb/cJ donor mice after lethal irradiation. Syngeneic mice were then given (or not) azacytidine (AZA, 0.5 mg/kg or 2 mg/kg) administered subcutaneously every 48h from day +10 to day +30. (A-B) FACS analyses showing Treg frequencies in blood (A) and spleen at day +52 (B) with an increased Treg frequency in blood at days +35 and +49 after transplantation for the highest dose of AZA-treated mice and a higher Treg frequency in spleen at day +52. (C-D) Demonstration of an increased frequency of activated Tregs (CD103^+^) in blood (C) at day +49 and in spleen at day +52 (D) for the highest dose of AZA.

**
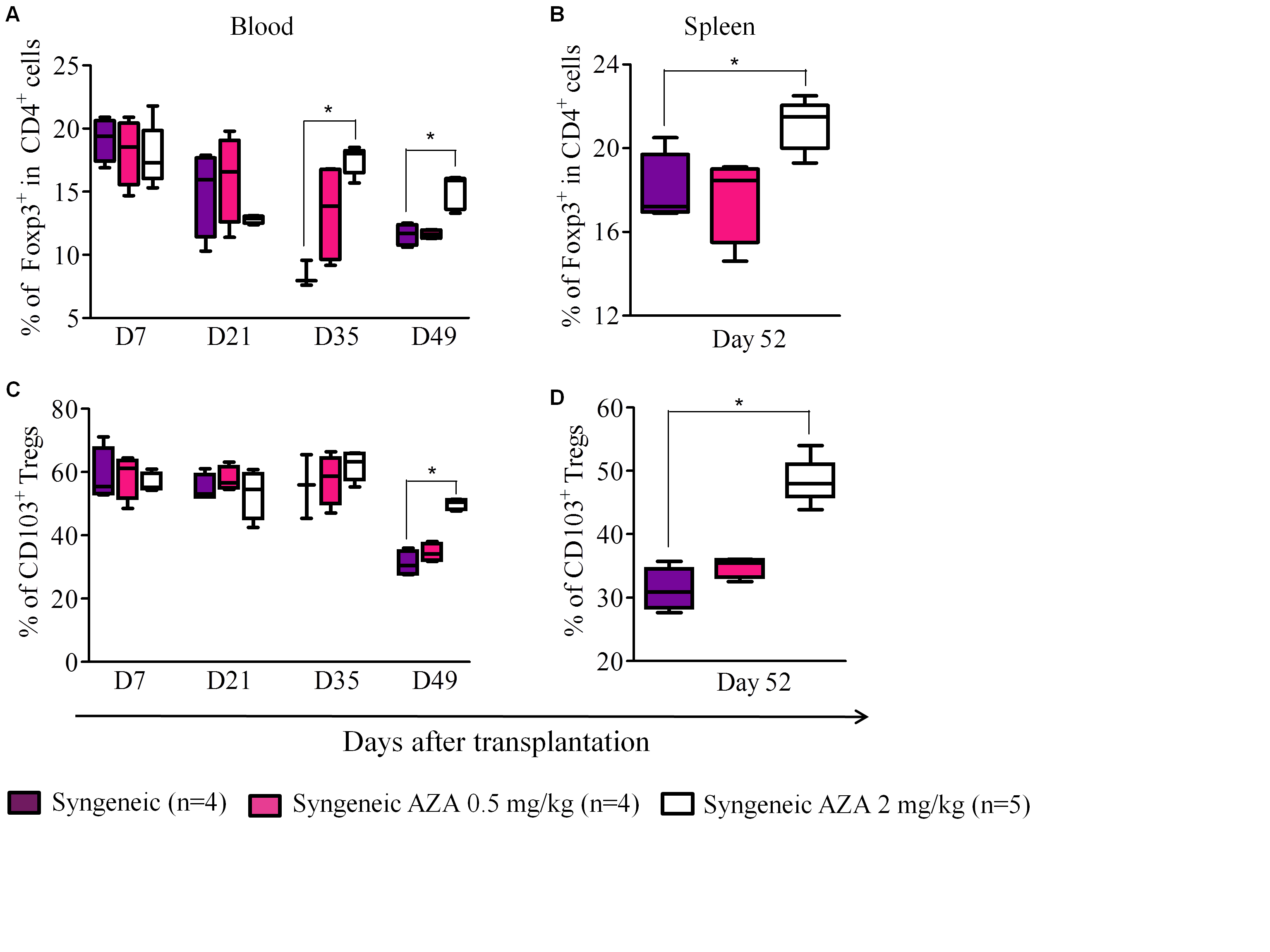
**

**Supplemental Figure 5. Azacytidine does not induce the generation of CD8^+^FoxP3^+^ regulatory T cells after allogeneic or syngeneic transplantation.**

Balb/cJ mice were injected i.v. with 10.10^6^ bone marrow cells and 70.10^6^ splenocytes from B10.D2 (allogeneic) or Balb/cJ (syngeneic) donor mice after lethal irradiation. Mice were then given (or not) azacytidine (AZA, 0.5 mg/kg or 2 mg/kg) administered subcutaneously every 48h from day +10 to day +30. The figure shows representative dot plots demonstrating the absence of CD8^+^Foxp3^+^ regulatory T cells in AZA-treated mice on days +35 and +49 (in contrast to CD4^+^Foxp3^+^ regulatory T cells) both in the allogeneic (A-D) or in the syngeneic (E-H) setting. Populations were gated on total CD8^+^ T cells (A-C-E-G) for CD8^+^FoxP3^+^ cells. For regulatory CD4^+^FoxP3^+^ and conventional CD4^+^Foxp3- T cells, populations were gated on total CD4^+^ T cells (B-D-F-H).

**
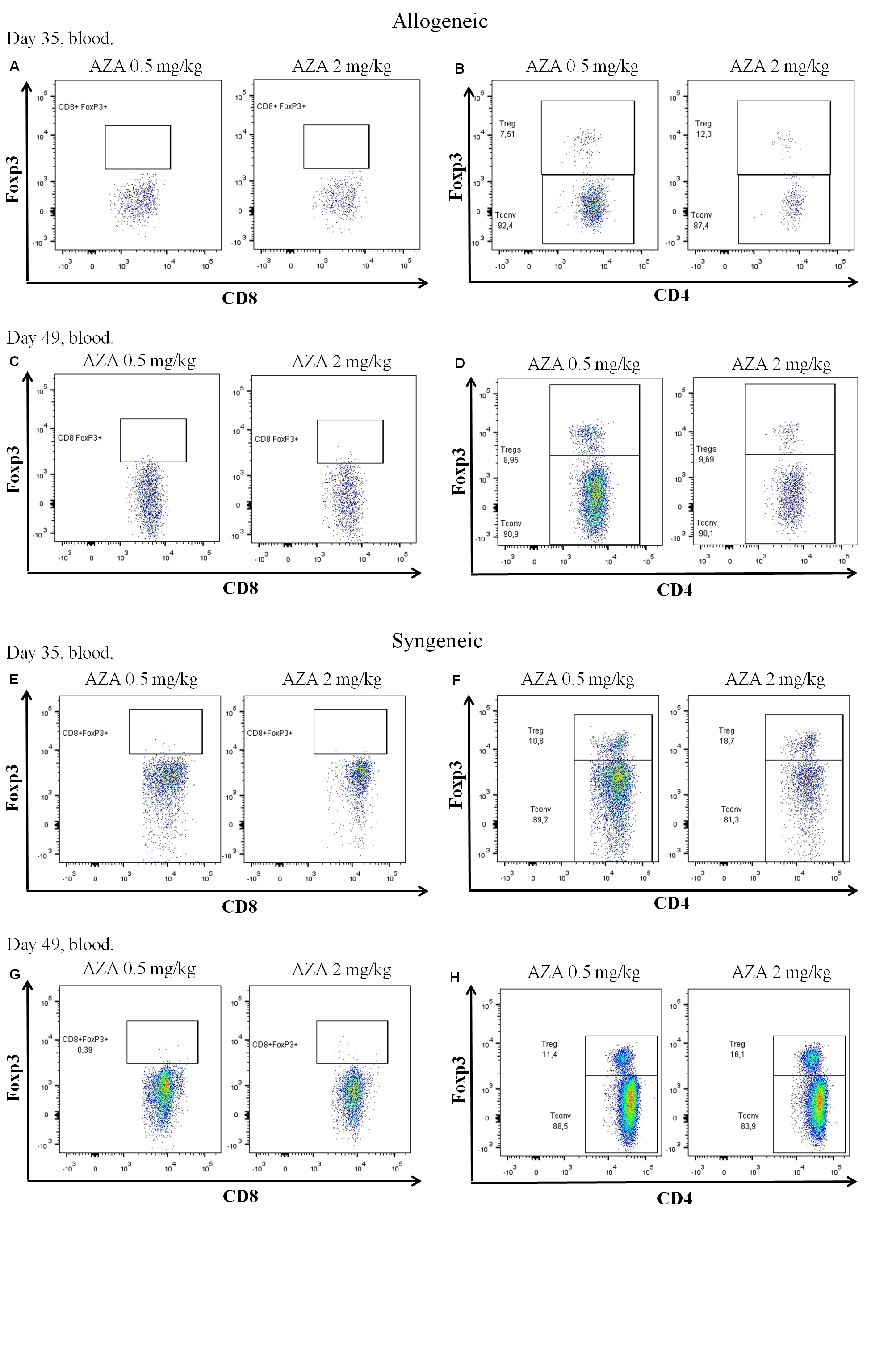
**

**Supplemental Figure 6: Azacytidine does not restrict T-cell Vβ (TCR Vβ) repertoire diversity on day +35 and +52**

Balb/cJ mice were injected i.v. with 10.10^6^ bone marrow cells and 70.10^6^ splenocytes from B10.D2 donor mice after lethal irradiation. Mice were then given (or not) azacytidine (AZA, 2 mg/kg), administered subcutaneously every 48h from day +10 to day +30. RNA was extracted from the spleens of four control and four AZA-treated mice on day +35 (A), or from the spleens of two control and two AZA-treated mice on day +52 (B). Spectratyping CDR-3 analyses were then realized in order to assess the impact of AZA on TCR Vβ diversity.


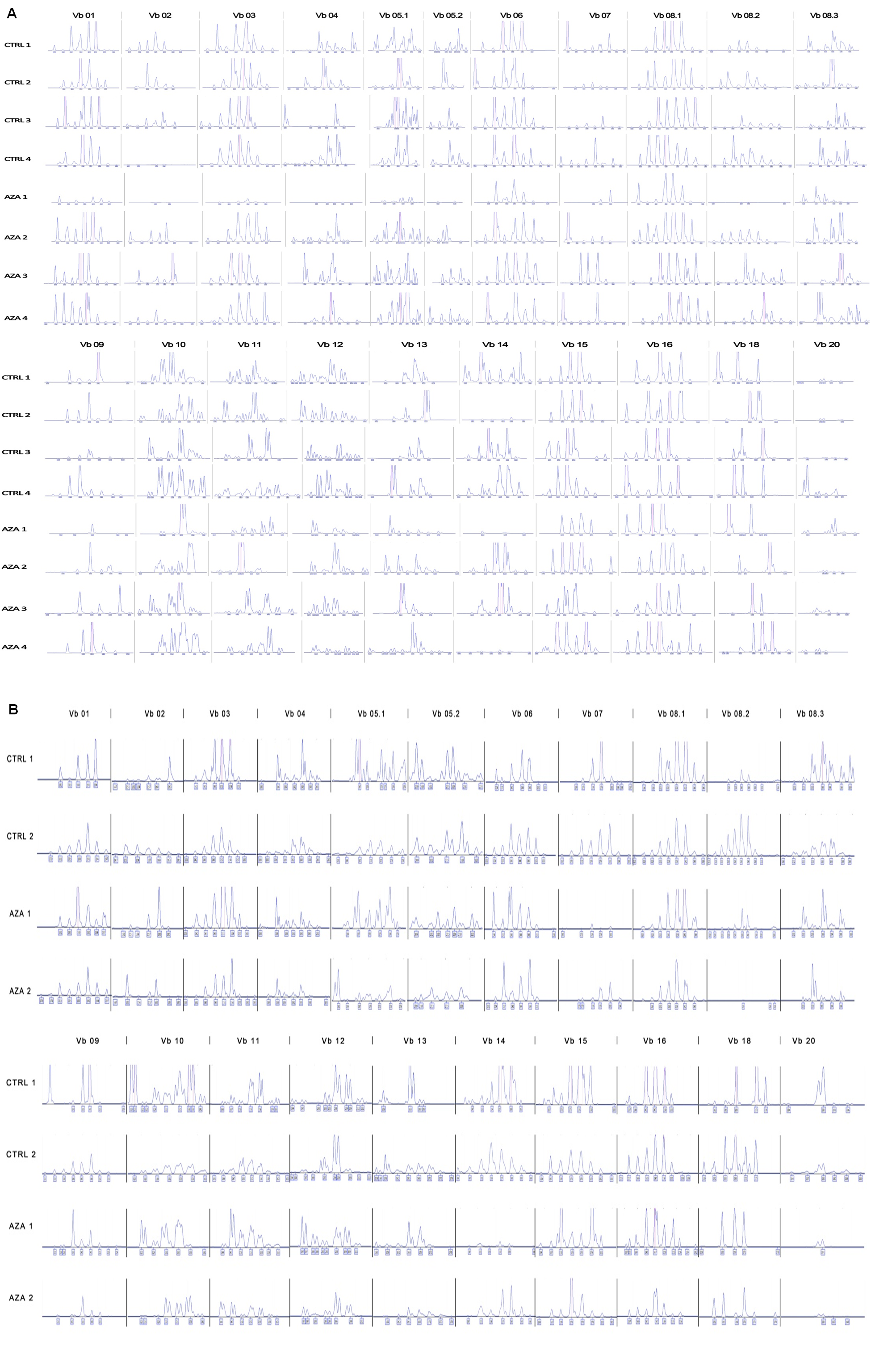

Supplement: Additional file 1: Figure S1. — A): Azacytidine administered every fourth day failed to prevent cGVHD. (B) Decitabine administered every other day ameliorated cGVHD. Figure S2: Impact of Azacytidine on blood counts and immune recovery after syngeneic transplantation. Figure S3: Impact of decitabine on skin, lungs and immune recovery. Figure S4: Azacytidine enhances Treg frequency and functions after syngeneic transplantation. Figure S5: Azacytidine does not induce the generation of CD8+FoxP3+ regulatory T cells after allogeneic or syngeneic transplantation. Figure S6: Azacytidine does not restrict T-cell Vβ (TCR Vβ) repertoire diversity on day +35 and +52. (DOCX 10679 kb) [file 13045_2016_281_MOESM1_ESM.docx]
